# Resolution of antibody in autoimmune urticaria

**DOI:** 10.1186/1710-1492-7-S2-A35

**Published:** 2011-11-14

**Authors:** Chrystyna Kalicinsky, Shamim Wadiwall

**Affiliations:** 1 University of Manitoba, Canada

## 

Chronic urticaria, defined as widespread daily or nearly daily wheals for at least 6 weeks, with or without angioedema, impacts on patients’ quality of life. Natural course is self-limited, with spontaneous remissions and occasional relapses. Antihistamines, leukotriene inhibitor, immunosuppressive agents are used. 60% are idiopathic and 40% are autoimmune due to presence of anti-IgE antibody or IgG autoantibodies against Fc∑R1. An association exists between chronic urticaria and autoimmune diseases. We report a case of a patient with autoimune urticaria, thyroid disease and vitiligo, who showed resolution of histamine releasing antibody (reflab) on remission.

In February 2005, a 44 yr old healthy woman was referred to the Allergy outpatient clinic with two month history of daily hives, moderately controlled with antihistamines, with good response to oral steroid. Lesions were pruritic, raised, erythematous, lasting for < 24 hrs and resolved with purplish discoloration. Screening negative for malignancy, connective tissue disease. TSH normal. Histamine releasing antibody positive, maximum histamine release 27% (<16%). Skin biopsy confirmed chronic urticaria with neutrophils without vasculitis. Sulfasalazine was not tolerated, but control attained with antihistamines and leukotriene inhibitor. By one year, she failed trial of weaning medications.

6 months after presentation she developed vitiligo. 5 years later, she was hypothyroid. By 3 years, the urticaria was in complete remission without medications. Repeat histamine releasing antibody was negative.

There is a known association of severe chronic urticaria with auto antibody etiology and other autoimmune disease. Does resolution of antibody correlate with achieving remission? Further prospective studies are required to establish this relationship.

